# Mitochondrial might: powering the peripartum for risk and resilience

**DOI:** 10.3389/fnbeh.2023.1286811

**Published:** 2023-12-20

**Authors:** Erin Gorman-Sandler, Gabrielle Wood, Nazharee Cloude, Noelle Frambes, Hannah Brennen, Breanna Robertson, Fiona Hollis

**Affiliations:** ^1^Department of Pharmacology, Physiology, and Neuroscience, University of South Carolina School of Medicine, Columbia, SC, United States; ^2^Columbia VA Healthcare System, Columbia, SC, United States; ^3^USC Institute for Cardiovascular Disease Research, Columbia, SC, United States

**Keywords:** stress, peripartum, postpartum, resilience, mitochondria, maternal, depression, pregnancy

## Abstract

The peripartum period, characterized by dynamic hormonal shifts and physiological adaptations, has been recognized as a potentially vulnerable period for the development of mood disorders such as postpartum depression (PPD). Stress is a well-established risk factor for developing PPD and is known to modulate mitochondrial function. While primarily known for their role in energy production, mitochondria also influence processes such as stress regulation, steroid hormone synthesis, glucocorticoid response, GABA metabolism, and immune modulation – all of which are crucial for healthy pregnancy and relevant to PPD pathology. While mitochondrial function has been implicated in other psychiatric illnesses, its role in peripartum stress and mental health remains largely unexplored, especially in relation to the brain. In this review, we first provide an overview of mitochondrial involvement in processes implicated in peripartum mood disorders, underscoring their potential role in mediating pathology. We then discuss clinical and preclinical studies of mitochondria in the context of peripartum stress and mental health, emphasizing the need for better understanding of this relationship. Finally, we propose mitochondria as biological mediators of resilience to peripartum mood disorders.

## 1 Introduction

### 1.1 Mitochondria are mediators of stress resilience

Stress exposure is a major risk factor for numerous pathologies, including major depressive disorder and anxiety. However, data from both preclinical and clinical studies highlight individual differences in stress response that result in resilient and susceptible phenotypes. The mechanisms underlying the development of these phenotypes have been a major research focus in the last decade or so, with studies identifying roles for epigenetic, genetic, and gene-by-environment interactions (Elbau et al., 2019). Interestingly, a number of biological processes implicated in individual differences in stress response converge onto the mitochondrion, resulting in a growing literature surrounding the role of mitochondrial function in stress-induced pathologies.

Mitochondria are bioenergetic, subcellular organelles known primarily for their role in energy production (in the form of adenosine triphosphate; ATP) through oxidative phosphorylation (OXPHOS). However, mitochondria have risen over the last two decades as crucial stress targets and mediators, contributing to psychiatric health and illness (Picard et al., 2016, 2018). The brain is heavily reliant on mitochondria for energy, consuming a disproportionate amount of energy for its size (20% of the body’s total energy) (Clarke, 1999), and is sensitive to even slight reductions in mitochondrial activity. Stress exposure significantly increases the brain’s energy needs (Bryan, 1990) and chronic exposure can exhaust spare energy reserves, leading to allostatic load/overload and an increased risk for pathology (Bobba-Alves et al., 2022). It is becoming increasingly clear that mitochondria are interconnected at multiple layers of the stress response and their function in the face of stress is critical to adaptation. In addition to increasing production of ATP to swiftly respond to increased energy demands (Picard et al., 2014), mitochondria house the rate-limiting enzymes required for the synthesis of glucocorticoids that are released in response to stress (Miller, 2013). Additionally, mitochondria have receptors for glucocorticoids on their outer membrane, allowing them to sense and respond to stress, as well as receive effects of stress (Psarra et al., 2006). Indeed, stress has significant effects on mitochondrial quality and function, resulting in the phenomenon known as mitochondrial allostatic load (Picard et al., 2014, 2018; Bobba-Alves et al., 2022). A growing number of studies have found that chronic stress reduces mitochondrial respiration, gene, and protein expression (Du et al., 2009; Gong et al., 2011; Hunter et al., 2016; de Souza Mota et al., 2017). Moreover, altered mitochondrial function as a result of stress exposure has been linked to behaviors associated with major depressive disorders and anxiety discussed in several notable reviews (Allen et al., 2018; Picard et al., 2018; Filiou and Sandi, 2019; Daniels et al., 2020; Morató and Sandi, 2020).

Notably, studies have identified mitochondrial function as a key aspect of stress resilience (Morava and Kozicz, 2013; Morató and Sandi, 2020; van der Kooij, 2020; Weger et al., 2020). While substantial progress has been made to understand the effects of stress on mitochondrial function, the vast majority of studies have been performed in adult male subjects. As females exhibit significantly higher proportions of neuropsychiatric disorders such as depression (Galea and Parekh, 2023), the knowledge gap surrounding potential sex differences in mitochondrial health is problematic. Additionally, there is a dearth of information surrounding the effects of stress on mitochondrial function across the lifespan. Chronic stress at any point can be deleterious, however, studies have identified key windows across development that are more sensitive to stress – and therefore potential targets to enhance resilience. Of particular interest is the peripartum period, which has been suggested as a period of vulnerability for the development of neuropsychiatric disorders (Vesga-Lopez et al., 2008). Pregnancy and the peripartum are particularly dramatic periods of physical and physiological adaptation where drastic hormonal fluctuations, driven primarily by the placenta, occur to ultimately promote placental growth, pregnancy maintenance, maternal behavior, and the healthy development of offspring (Bridges et al., 2008; Burton and Fowden, 2015). These fluctuations, even when resolved, contribute to both short- and long-term consequences on maternal and offspring health. As the majority (approximately 80%) of women have experienced pregnancy and parturition at least once (Martinez et al., 2018), there is an urgent need to understand the short- and long-term consequences of peripartum experiences. In this review, we briefly highlight the peripartum as a period of stress susceptibility and examine the evidence for mitochondrial involvement in peripartum stress resilience. We identify open questions and propose mitochondrial function as a potential area for resilience research in females – an understudied vulnerable population.

### 1.2 Pregnancy as a critical window of stress vulnerability

Females experience several unique biological events across the lifespan that are characterized by distinct physiological changes. These periods of the lifespan (i.e., puberty, menstruation, pregnancy/motherhood, menopause) are associated with increased vulnerability to the development of stress-related disorders such as depression (Lokuge et al., 2011; Deems and Leuner, 2020). This is evident by the disproportionate amount of females who experience these disorders compared to males (24% vs. 13.3%, respectively) (Lee, 2023). However, pregnancy and the postpartum period are characterized by widespread and extensive physiological changes that are incurred in the process of supporting the development of the fetus. These changes include fluctuations in hormones, as well as increased plasma volume, cardiac output, metabolic rate, and fluctuating immune, and neurobiological systems (Fahey and Shenassa, 2013). During pregnancy, placental hormone production largely induces dramatic fluctuations of steroid hormones at levels typically not observed at any other point in a healthy women’s life. Estrogens such as estradiol (E2) and estriol (E3) are increased 300 and 1,000 times, respectively; glucocorticoids are secreted 2–3 times higher, and progesterone (P4) is nearly 20 times higher compared to non-pregnant women (Adamcová et al., 2018; Deems and Leuner, 2020). Following parturition and expulsion of the placenta, estrogen and P4 rapidly decline (Hendrick et al., 1998), while glucocorticoids remain elevated compared to pre-pregnancy levels (Jung et al., 2011). Additionally, immune system alterations have been noted in both pregnancy and the postpartum period. That is, while normal pregnancy is characterized by an anti-inflammatory state with proinflammatory shifts according to pregnancy stage (Bränn et al., 2019), stress during pregnancy can alter the immunological profile of mothers, resulting in a more pro-inflammatory state that is associated with peripartum mood disorders (Anderson and Maes, 2013; Christian, 2015; Dye et al., 2022).

Finally, the brain undergoes significant and long-lasting changes in structure, function, and neural/glial plasticity during the peripartum period (see excellent reviews, Oatridge et al., 2002; Brunton and Russell, 2008; Hillerer et al., 2014; Hoekzema et al., 2017; Barba-Müller et al., 2019; Cárdenas et al., 2019; Puri et al., 2023; Servin-Barthet et al., 2023). The net result is often one of decreased volume, activation, neurogenesis, glial density and numbers (Pawluski et al., 2016; Haim et al., 2017; Hoekzema et al., 2017). The purpose of these alterations is unclear, with some researchers postulating these changes as positive refinements that are necessary for the transition to motherhood (Pawluski et al., 2022) and others suggesting a tradeoff such that essential cognitive functions for motherhood are prioritized at the expense of other functions (Barda et al., 2021). These two hypotheses are not mutually exclusive (Orchard et al., 2023), and they highlight the potential for impacts to mental health if parity-induced neurobiological changes go awry.

In addition to changes in physiology, the peripartum period is characterized by increased levels of social, financial, emotional, and physical stress. Anxiety or fear of the birthing experience is a common pregnancy-specific stressor (Alehagen et al., 2006; Hall et al., 2009). Sleep deprivation, pain, as well as emotional, cognitive, and financial demands following childbirth are common postpartum-specific stressors (reviewed in Cárdenas et al., 2019). Nearly half of women report experiencing at least one stressor during pregnancy, and 20% report experiencing at least three, with stress exposure dose-dependently corresponding to maternal morbidity (Stone et al., 2015; Lawrence et al., 2022). Women who experienced seven or more stressors had higher incidences of postpartum depression (PPD) (Lawrence et al., 2022), with stress exposure counting as one of the largest risk factors for PPD, after a prior history of depression (Stone et al., 2015). Even the perception of stress can interact with the peripartum period to influence mood, as postpartum women who reported an increase in the perception of stress in the 2 years following birth also reported increased depressive symptoms (Leonard et al., 2020). Preclinical studies in female rodents support a role for peripartum stress in the development of neuropsychiatric disorders such as PPD (summarized in Table 1). Exposure to stress before (Gemmel et al., 2018; Czarzasta et al., 2019) or, during gestation (Pardon et al., 2000; Smith et al., 2004; Misdrahi et al., 2005; O’Mahoney et al., 2006; Haim et al., 2014; Leuner et al., 2014; Gemmel et al., 2016; Gifford et al., 2023; Gorman-Sandler et al., 2023) or, in the postpartum period during lactation (Nephew and Bridges, 2011; Medina et al., 2021; Cruz et al., 2023) in the postpartum period during lactation, increases the expression of PPD-relevant behaviors including anhedonia and disrupted maternal care. Similarly, treating female rats with the steroid stress hormone, corticosterone, is sufficient to elicit anhedonia and decreased maternal care (Brummelte et al., 2006; Pawluski et al., 2009; Brummelte and Galea, 2010; Workman et al., 2013, 2016). Given the links between stress exposure and mood disorders, it is clear that stress resilience during the peripartum period is imperative for maternal mental health.

**Table 1:** Preclinical peripartum stress models.

**Species/Strain**	**Stress type**	**Stress timing and duration**	**Behavioral outcomes**	**References**
Mouse/B6D2F1	Chronic ultramild stress	3 weeks beginning from time of mating to PD0; cage tilt, confinement to small cage, paired housing, one overnight period of difficult access to food, one overnight period of light exposure, and one overnight period in a soiled cage	↓ aggression towards intruder	Pardon et al. (2000)
Rat/Hooded Lister	Chronic restraint stress	1h daily from GD 10-20	↑ immobility in second exposure to FST, ↓ maternal care	Smith et al. (2004)
Mouse/B6D2F1	Chronic ultramild stress	From GD 1-15 or PD 1-3; cage tilt, confinement to small cage, paired housing, one overnight period of difficult access to food, one overnight period of light exposure, and one overnight period in a soiled cage	No behavioral outcomes measured	Misdrahi et al. (2005)
Rat/Sprague-Dawley	Chronic subcutaneous CORT injections	40 mg/kg CORT injections daily from PD 1-26	↓ maternal care and ↑ immobility in FST	Brummelte et al. (2006)
Rat/Fisher	Chronic restraint stress	3X 45-min bouts daily from GD 14-21	↑ immobility in FST	O’Mahoney et al. (2006)
Rat/Sprague-Dawley	Chronic subcutaneous CORT injections	Low (10 mg/kg) or high (40 mg/kg) concentrations of CORT injected daily from GD 10-20 and/or PD 2-24	↓ maternal care and ↑ immobility in FST in high CORT postpartum dams	Brummelte and Galea (2010)
Rat/Sprague-Dawley	Chronic social stress	Daily presentation of novel male intruder for 1h from PD 2-16	↓ maternal care, ↑ agression and ↑ self-grooming	Nephew and Bridges (2011)
Rat/Sprague-Dawley	Chronic subcutaneous CORT injections and impoverished housing	40 mg/kg CORT injected daily from PD 2-24 (housing conditions concurrent with injections)	↓ maternal care and ↑ immobility in FST	Workman et al. (2013)
Rat/Sprague-Dawley	Chronic inescapable swim stress and restraint stress	20 min of inescapable swim stress 2X daily from GD 7-13, then restraint 2X daily for 30 min from GD 14-20 (exception: restraint was used to replace inescapable swim stress for rats subjected to FST)	↑ immobility in FST, ↓ maternal care, ↓ cognitive flexibility in PFC-mediated attentional set shifting task	Leuner et al. (2014)
Rat/Sprague-Dawley	Chronic restraint stress	2X 30-min bouts daily from GD 7-20	↑ immobility in FST at early/mid and late postpartum	Haim et al. (2014)
Rat/Sprague-Dawley	Chronic subcutaneous CORT injections	40 mg/kg CORT injected daily from PD 2-23	↓ maternal care, ↑ self-grooming, ↑ immobility in FST	Workman et al. (2016)
Rat/Sprague-Dawley	Chronic restraint stress	3X 45-min bouts daily from GD 15-20, and 2X on GD21	No behavioral outcomes measured	Gemmel et al. (2016)
Rat/Sprague-Dawley	Chronic unpredictable stress	0-2 stressors/day for 3 weeks prior to breeding; restraint under bright light, overcrowding, overnight damp bedding, 12h food deprivation; 5 min of forced swimming, cage rotation	↓ breeding success and maintenance of pregnancy, ↑ time nursing in first week postpartum	Gemmel et al. (2018)
Rat/Spague-Dawley	Chronic restraint stress	1h daily for 5 consecutive weeks prior to breeding	↓ self-grooming in splash test, ↓ time in center in OFT	Czarzasta et al. (2019)
Rat/Sprague-Dawley	Chronic variable stress	1 stressor/day from PD 2-21; wet bedding, 4h empty water bottle, overnight light exposure, tail pinch, dirty bedding, stroboscopic lighting, white noise, low bedding, and cage tilt	↑ distance traveled in the OFT	Medina et al. (2021)
Rat/Sprague-Dawley	Limited bedding and nesting	GD 19 to PD 9, with bedding replaced daily	↓ arched-back nursing and ↑ simple blanket nursing, ↓ consumption of novel candy in hoarding task, ↓ time in the open arms of EPM	Gifford et al. (2023)
Mouse/Swiss	Chronic offspring deprivation and obesity	3h daily from PD 1-10	↓ grooming after splash test	Cruz et al. (2023)
Rat/Wistar	Chronic mild unpredictable stress	1 stressor/day of varying lengths of time and times of day from GD 10-19; white noise, wet bedding, no bedding, cage tilt, overnight light exposure, and acute restraint stress	↓ sucrose preference, ↓ maternal care, ↓ latency to immobility in FST	Gorman-Sandler et al. (2023)

PD, postpartum day(s); GD, gestational day(s); FST, forced swim test; CORT, corticosterone; PFC, prefrontal cortex; OFT, open field test; EPM, elevated plus maze.

There are a number of mental health disorders that develop and/or are diagnosed in the peripartum period. Mood alterations are a common maternal health complication, with depressive symptoms reported in 70% of women during pregnancy (O’Hara and Wisner, 2014; Becker et al., 2016). Additionally, up to 20% of mothers experience severe depressive symptoms within the year following parturition, and are diagnosed with PPD (O’Hara and Swain, 1996; Haight et al., 2019). Postpartum anxiety (PPA) is another maternal mood disorder with estimates pointing to a 20.7% prevalence in either pregnancy or postpartum periods (Fawcett et al., 2019) and a comorbidity with PPD ranging from 3% to 6% (Farr et al., 2014). Postpartum psychosis is a dangerous condition that is characterized by thoughts or acts of self-harm or harm to the child. While rarer than PPD or PPA with a 0.12%–0.25% prevalence (VanderKruik et al., 2017), the severe consequences of this disorder make postpartum psychosis a significant public health issue. Finally, a small but significant percentage of women experience a traumatic childbirth experience that gives rise to posttraumatic stress disorder or posttraumatic stress symptoms (Schobinger et al., 2020; Heyne et al., 2022). Recent estimates place the prevalence of birth-related posttraumatic stress disorder at 4.7%, and 12.3% for birth-related posttraumatic stress symptoms (Heyne et al., 2022), though rates are increased in high-risk populations (e.g., caesarean section) (Schobinger et al., 2020). Taken together, it is no surprise that the peripartum period is considered a period when women are most at risk for the development of neuropsychiatric disorders.

## 2 Mitochondrial involvement in physiological adaptations and psychiatric disorders of the peripartum

**Figure 1:**
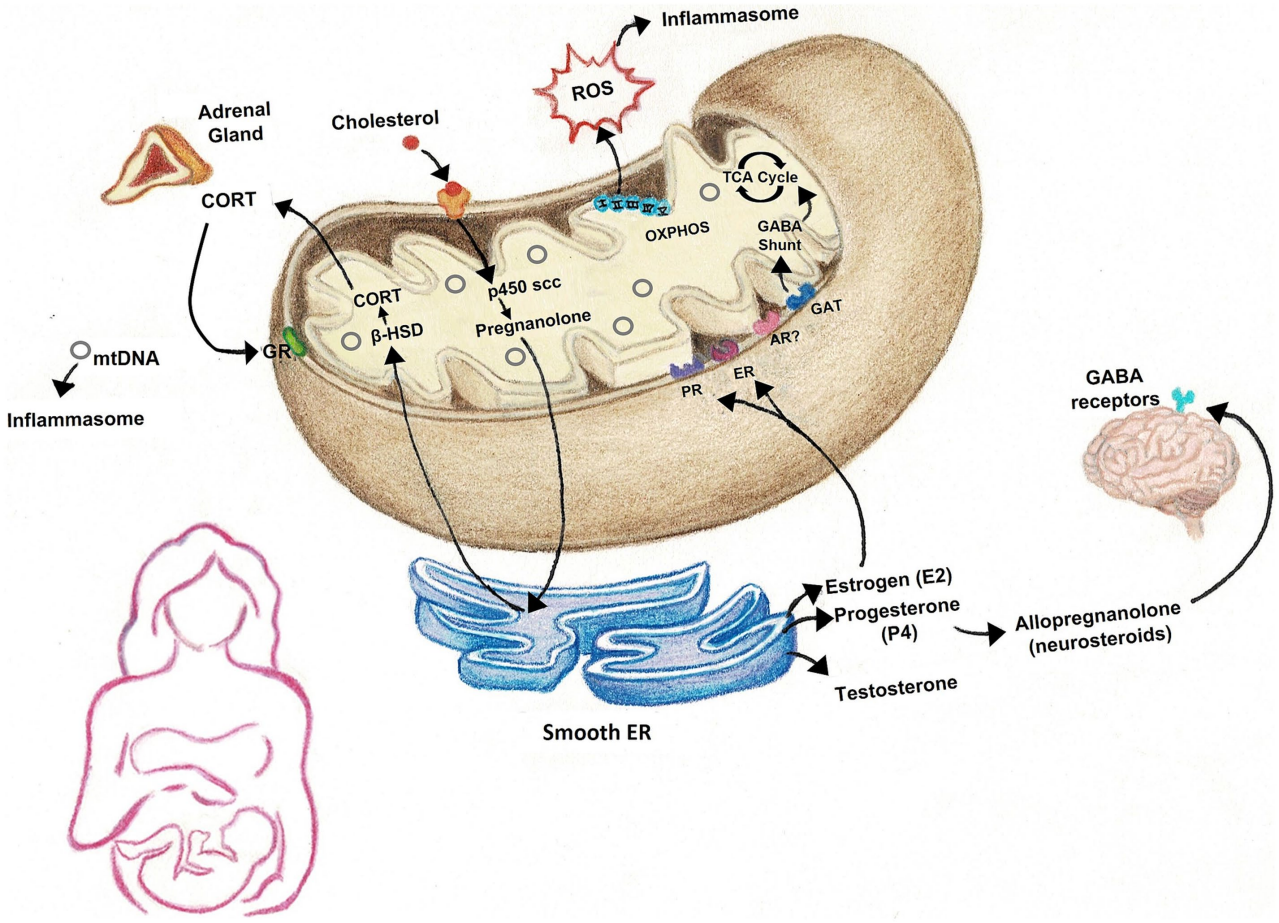
Many of the physiological processes that occur in the peripartum rely on mitochondria. Mitochondria house the rate-limiting enzymes responsible for the synthesis of steroid hormones that fluctuate across the peripartum, including gonadal hormones, glucocorticoids, and neurosteroids, such as allopregnanolone. The electron transport chain and ATP synthase are responsible for the production of energy in the form of ATP, but also generate reactive oxygen species (ROS), which, along with cell-free mtDNA, can activate inflammasomes to promote inflammation. ROS may also have roles in cell signaling. GABA levels are also reported to fluctuate across the peripartum period and mitochondria are important sites of GABA catabolism. CORT, corticosterone/cortisol; ROS, reactive oxygen species; mtDNA, mitochondrial DNA; GR, glucocorticoid receptor; β-HSD, beta-hydroxysteroid dehydrogenase; p450 scc, cholesterol side-chain cleavage enzyme; OXPHOS, oxidative phosphorylation; TCA cycle, tricarboxylic acid cycle; GABA, gamma-aminobutyric acid; PR, progesterone receptor; ER, estrogen receptor; AR, androgen receptor; GAT, GABA transporter.

Many studies have focused heavily on energy production when examining the role of mitochondria in medicine. While mitochondrial ATP production is crucial in cell survival, there are numerous other functions of mitochondria that are particularly linked to physiological changes that occur across the peripartum period (Figure 1). Thus mitochondrial function has great potential to mediate postpartum-induced alterations in brain and behavior and may contribute to susceptibility in the development of mood disorders.

### 2.1 Steroid hormones

Mitochondria are crucial for the synthesis of the very same steroid hormones that fluctuate in the peripartum period and have been implicated in postpartum pathologies (Miller, 2013). First, all steroid hormones are derived from cholesterol, which is delivered to the inner mitochondrial membrane by cholesterol transport proteins such as translocator protein (18 kDa) (TSPO) (Miller and Bose, 2011). The rate-limiting enzyme, cytochrome P450 cholesterol side-chain cleavage enzyme, is housed within the inner mitochondrial membrane and converts cholesterol to pregnanolone (Hu et al., 2010; Papadopoulos and Miller, 2012). Additional downstream reactions by mitochondrial enzymes result in synthesis of glucocorticoids, estrogens, progesterone, allopregnanolone, and testosterone in a tissue-specific manner (Miller, 2013). Moreover, receptors for glucocorticoids, estrogens, and progesterone have been observed on the outer mitochondrial membrane, allowing these steroid hormones to bind and suggesting direct influence on mitochondrial respiration to meet energy needs in response to their associated growth and developmental processes (Psarra et al., 2006).

Indeed, glucocorticoids have been shown to exert biphasic effects on mitochondrial function, with high concentrations inducing deleterious effects including apoptosis and altered gene transcription (Du et al., 2009; Hunter et al., 2016). Additionally, disruptions in mitochondrial function have been shown to significantly alter both hypothalamic–pituitary–adrenal (HPA), and sympathetic adrenal-medulla axes activation, shaping the response to stress (Picard et al., 2015). Thus, elevated glucocorticoids can induce mitochondrial dysfunction, which can then lead to either exaggerated or blunted stress responses. Interestingly, abnormal glucocorticoid responses during pregnancy and early postpartum have been hypothesized as a contributing and possibly predicting factor of peripartum mood disorders, such as PPD (Hillerer et al., 2012; Dickens and Pawluski, 2018). While the literature is conflicted (Rich-Edwards et al., 2009; Meltzer-Brody et al., 2011; Payne and Maguire, 2019), several studies support this hypothesis, reporting that women who exhibited altered or blunted cortisol patterns during the peripartum period were at greater risk of PPD (Bloch et al., 2003; Scheyer and Urizar, 2016). Moreover, preclinical models employing glucocorticoid injection support this hypothesis with reports of PPD-relevant behaviors such as anhedonia and disrupted maternal care (Brummelte and Galea, 2010). Unfortunately, it is unclear how peripartum glucocorticoid treatment affects mitochondria, as studies of mitochondrial respiration, content, and/or internalization of steroid receptors in these preclinical models are so far absent. The sustained elevations in glucocorticoids induced by normal pregnancy may result in altered mitochondrial functions that are unable to properly respond to further stress exposure, potentially leading to pathology. Additionally, alterations in cholesterol transport into mitochondria and subsequent steroidogenesis may contribute to HPA axis dysregulation observed in PPD and PPD-like phenotypes. Two separate groups explored the therapeutic potential of TSPO ligands on PPD-relevant phenotypes, induced by hormone-stimulated pregnancy and estradiol withdrawal in mice (Li et al., 2018) and rats (Ren et al., 2020), demonstrating a role for mitochondrial import machinery on HPA axis signaling and depressive- and anxiety-like behaviors in the postpartum.

Sex hormones such as estrogens and progesterone can also bind to receptors on mitochondria and modulate function. E2 is the predominant estrogen in circulation, though E3 is significantly increased during pregnancy (Robinson and Klein, 2012). In general, estrogens are thought to exert a protective role on mitochondria through both genomic and non-genomic mechanisms (Klinge, 2020). E2 affects stress responses of the HPA axis (Handa et al., 2012) and has been shown to benefit mitochondrial function by reducing oxidative damage and promoting energy production via OXPHOS (Brinton, 2008). Estrogen receptors can localize to mitochondria, where their actions can affect respiratory activity, mitochondrial enzymatic activity, reduce lipid peroxidation, and alter mitochondrial dynamics (Yang et al., 2004, 2009; Irwin et al., 2008; Beikoghli Kalkhoran and Kararigas, 2022). It is possible that the fluctuation of estrogen levels during and shortly following pregnancy have downstream effects on mitochondrial function, promoting a dysfunctional phenotype. Specifically in the brain, studies in ovariectomized rats with E2 and P4 replacement observed significantly increased brain mitochondrial function, decreased lipid peroxidation and reactive oxygen species (ROS), and increased respiratory control ratios relative to oil-treated controls (Irwin et al., 2008). In peripheral cells, genetic manipulations where estrogen receptors were reduced or depleted resulted in significant reductions in mitochondrial respiration, mitochondrial DNA (mtDNA) turnover, and reserve (Salama et al., 2014; Ribas et al., 2016). Interestingly, a recent study reported significantly decreased ERα, but not ERβ, expression at the gene and protein levels in the medial preoptic area of postpartum rats compared to nonpregnant controls (Gifford et al., 2023), pointing to a significant effect of pregnancy alone.

The literature is mixed regarding the effects of parturition on progesterone receptors in hypothalamic nuclei, with reports of increased (Francis et al., 2002), decreased (Carrillo-Martínez et al., 2011), and no change (Numan et al., 1999) in postpartum expression. However, studies have implicated sex hormone levels in peripartum mood disorders, with changes in E2 and P4 associated with PPD (Bloch et al., 2000). In fact, hormone-stimulated pregnancy and estradiol withdrawal is also used as a preclinical model to simulate the dramatic fluctuations that occur during the peripartum, resulting in PPD-relevant behaviors (Perani and Slattery, 2014). Although sex hormone effects on mitochondria have been reported (Klinge, 2020), effects during specific peripartum periods and following peripartum stress have been understudied. Moreover, the levels of sex hormones within specific brain regions across the peripartum are also unclear, as techniques to sensitively quantify these hormones have only recently become available (Gravitte et al., 2021). Investigating the effects of decreased receptor expression and/or hormone levels in the postpartum period may reveal that protective mechanisms which promote mitochondrial function may be insufficient, leading to greater vulnerability to stress.

### 2.2 GABA and allopregnanolone

Mitochondria are also important sites of gamma-aminobutyric acid (GABA) catabolism (Ravasz et al., 2017). In addition to its role as a neurotransmitter, GABA also acts as a metabolic intermediary via a GABA shunt that increases tricarboxylic acid (TCA) cycle intermediates, such as succinate (Martin and Rimvall, 1993). Succinate is produced as part of the TCA cycle by an α-ketoglutarate-dependent pathway, however, the coordinated action of glutamate decarboxylase, GABA-transaminase and succinic semialdehyde dehydrogenase allow the production of succinate to bypass part of the TCA cycle to produce ATP (Cavalcanti-de-Albuquerque et al., 2022). Recent work has begun to delineate a role for the GABA shunt to function in mitochondrial redox metabolism to drive changes in behavior and metabolism (Parviz et al., 2014; Salminen et al., 2016; Ravasz et al., 2017; Cavalcanti-de-Albuquerque et al., 2022). In fact, the presence of GABA, in combination with α-ketoglutarate, enhanced mitochondrial respiration and ROS in brain regions relevant to maternal health such as the hypothalamus (Cavalcanti-de-Albuquerque et al., 2022). GABA levels fluctuate across the peripartum period, with studies showing lower levels in cerebrospinal fluid in late pregnancy (Altemus et al., 2004) and increases during labor (Sethuraman et al., 2006). The link between GABA levels and PPD is unclear as some studies in peripartum women found negative correlations between GABA levels and depression symptom severity level (Deligiannidis et al., 2016; Walton and Maguire, 2019), while others found increased levels in PPD (Epperson et al., 2006). Meanwhile, preclinical studies observed brain-region specific regulation of GABA release in response to pregnancy and offspring interaction (Lonstein et al., 2014).

GABA is also a target of neurosteroid actions, such as allopregnanolone (Puia et al., 1990; Majewska, 1992). Allopregnanolone is another metabolite of cholesterol – thus linking its synthesis to mitochondrial enzymes. Moreover, allopregnanolone has been shown to inhibit the mitochondrial permeability pore to confer neuroprotective effects (Sayeed et al., 2009) in addition to its actions as a positive allosteric modulator of GABA A-type receptors (GABA_A_R). Preclinical work identified significant decreases in GABAergic receptors in pregnant but not postpartum rats (Maguire and Mody, 2008). Additionally, mice lacking GABA_A_ δ-type receptors exhibited several PPD-relevant behaviors during pregnancy, suggesting a PPD-like endophenotype (Maguire and Mody, 2008). Treatment with a synthetic analog of allopregnanolone, Brexanolone, reversed PPD-like behaviors, including enhancing maternal care (Melón et al., 2018). During pregnancy, allopregnanolone concentrations increase, but then rapidly plummet following parturition, which has been shown to alter expression of GABA_A_R in specific brain regions (Walton and Maguire, 2019). Interestingly, clinical trials treating PPD patients with an allopregnanolone analog observed a striking therapeutic effect (Kanes S. J. et al., 2017; Kanes S. et al., 2017; Meltzer-Brody et al., 2018), leading to FDA approval of the first PPD therapeutics, Zulresso and Zurzuvae. However, the literature remains conflicted regarding the relationship between allopregnanolone and PPD, with inconsistent observations of allopregnanolone levels in PPD patients and an unclear mechanism by which allopregnanolone treatment alleviates PPD symptoms (Walton and Maguire, 2019; Paul et al., 2020).

Given the links between GABA, allopregnanolone, and mitochondrial function, one possibility is that allopregnanolone acts on GABA_A_R’s to consequently modulate mitochondrial respiration and energy production. An intriguing alternative hypothesis is that stress exposure during the peripartum period leads to altered levels of neurosteroids via induction of mitochondrial dysfunction. Rodents exposed to chronic stress exhibit reduced levels of allopregnanolone (Bali and Jaggi, 2014). Moreover, recent work found that chronic unpredictable stress significantly reduced neurosteroid levels and signaling in the amygdala, leading to depressive-like behaviors that were reversible by Brexanolone treatment (Antonoudiou et al., 2022; Walton et al., 2023). Conversely, allopregnanolone is associated with promoting a hypoactive HPA axis response to stressors (Brunton et al., 2014). It is therefore tempting to speculate that allopregnanolone treatment is effective because it acts downstream of a stress-induced mitochondrial deficit. The majority of studies have focused on GABA as either a neurotransmitter or, more recently, a target of neurosteroid signaling and there is a notable paucity of investigations into its role in metabolism in the peripartum period. Further studies expanding this focus will be crucial in understanding the relationship between GABA, allopregnanolone, and mitochondria.

### 2.3 Immune signaling and oxidative stress

Mitochondria are key participants in the activation of inflammasomes that lead to proinflammatory cytokine production (West and Shadel, 2017) via the release of ROS and other immunogenic compounds. Notably, mitochondrial ROS positively correlates with activation of inflammasomes linked to downstream release of interleukin (IL)-1β, IL-6, and other proinflammatory cytokines that are elevated in PPD (Nephew and Bridges et al., 2011; Zhou et al., 2011). Mitochondria are a major source of ROS however, their complexes are vulnerable targets of oxidative stress (Murphy, 2009). Complex I in particular is a major site of ROS production as well as target for detrimental ROS actions (Musatov and Robinson, 2012; Simeone et al., 2014). Oxidative damage to mitochondria can affect quality and function, eliciting further downstream inflammatory responses and ROS production. Consequently, oxidative stress and inflammation are frequently discussed in relation to each other. In the context of pregnancy, oxidative stress is increased, especially during the third trimester (Hung et al., 2010), and is likely related to the corresponding inflammatory signaling that occurs (Roomruangwong et al., 2018).

In addition to releasing ROS, the mitochondrion also releases its own DNA. Mitochondria contain a limited genome that is independent from the nuclear genome and housed in multiple copies of circular DNA structures bound in nucleoids (Lee and Han, 2017; Farge and Falkenberg, 2019). MtDNA can be found in most body fluids as cell-free mtDNA (*cf*-mtDNA) (reviewed in Trumpff et al., 2021). Recent work identified *cf*-mtDNA as a highly immunogenic compound capable of activating inflammasomes and downstream cytokine production due to the bacterial nature of its ancestry (Zhong et al., 2018). Interestingly, *cf*-mtDNA has been shown to increase with advancing gestational age and return to pre-pregnancy levels shortly after delivery, suggesting a fetal or placental source (Cushen et al., 2020; Bradshaw et al., 2022). Moreover, increased *cf*-mtDNA levels in pregnant women suffering from preeclampsia were found to induce higher levels of toll-like receptor 9 release compared to healthy counterparts (Williamson et al., 2019; Bradshaw et al., 2022). A preclinical study using a toll-like receptor 9 agonist found that stimulation resulted in a pregnancy-specific response (Goulopoulou et al., 2016), highlighting the potential for *cf*-mtDNA to induce unique effects in the peripartum period versus other developmental time points.

Exposure to stress may damage mitochondria, initiating the release of ROS or mtDNA, which further promotes a pro-inflammatory response via inflammasome activation and cytokine production (Zhou et al., 2011; Riley and Tait, 2020). Specifically, peripartum stress exposure may exacerbate *cf*-mtDNA levels as studies have shown significant increases in *cf*-mtDNA in serum of healthy individuals following acute stress (Hummel et al., 2018; Trumpff et al., 2019). Importantly, it should be noted that the role of *cf*-mtDNA as *uniquely* pro-inflammatory is not yet established as there are few causal studies and the potential for *cf*-mtDNA to function in cellular communication or other processes in addition to inducing inflammation should be considered (Trumpff et al., 2021). However, given the dynamic response of *cf*-mtDNA to environmental challenges, and its pro-inflammatory signaling capacity that may have pregnancy-specific effects, further functional studies of *cf*-mtDNA in the peripartum period are warranted. Mitochondrial ROS also has known cell-signaling actions and crucial roles in physiological processes including cell differentiation and oxygen sensing (Scialò et al., 2017; Sinenko et al., 2021). Thus, care should be taken with interpretation of findings to explore mtDNA and mtROS release within the context of cellular networks and physiological processes.

### 2.4 Oxytocin

Oxytocin (OXT) is a neuropeptide synthesized in the hypothalamus, with important roles during the peripartum (Froemke and Young, 2021). In addition to influencing parturition, nursing, and maternal behaviors such as maternal–infant attachment (Lévy et al., 1992; Nishimori et al., 1996; Walter et al., 2021), OXT also has suppressive effects on HPA axis reactivity, and in turn may protect against the physical and psychological stress associated with gestation and the peripartum (Bell et al., 2014). Given its roles in peripartum adaptations and stress, OXT has also been implicated in PPD (Post and Leuner, 2019; Thul et al., 2020; Levin and Ein-Dor, 2023). For example, one group assessed genetic and epigenetic variations in the OXT receptor gene in pregnant women who later developed PPD, showing that those with the rs53576_GG polymorphism and high methylation of the OXT receptor gene were three times more likely to develop PPD than those with a different allele (Bell et al., 2015). Another study demonstrated that lower oxytocin levels in plasma of pregnant individuals are associated with higher incidence of PPD symptoms (Skrundz et al., 2011).

OXT effects on mitochondria are less clear, especially within the brain, though studies suggest that OXT impacts mitochondrial membrane potential, activity, ATP levels, and ROS production (Gravina et al., 2011; Kaneko et al., 2016; Amini-Khoei et al., 2017). Notably, only one study to date links mitochondrial function with OXT in relation to peripartum stress. In a cohort of postpartum women evaluated for childhood maltreatment load, there were no differences in plasma OXT between those with high or low maltreatment (Boeck et al., 2018). However, in women with high maltreatment load only, there was a negative correlation between mitochondrial respiration in immune cells and plasma OXT, where lower OXT correlated with higher respiration (Boeck et al., 2018). These data suggest a potential protective mechanism of OXT on CM-related effects on respiration.

Unlike steroid hormones, the localization of oxytocin receptors to mitochondria has not yet been established (Bordt et al., 2019), and thus their observed effects on mitochondrial parameters are most likely indirect. For example, OXT induces increases in intracellular calcium that elicit downstream effects on mitochondrial membrane potential and ATP synthase activity (Gravina et al., 2011). In addition to classically studied effects on natural peripartum adaptations, OXT also has anti-inflammatory, antioxidant, and subsequently neuroprotective effects (Wang et al., 2015; Kamrani-Sharif et al., 2023). In light of the relationship already described between mitochondria and inflammatory/oxidative pathways (Section 2.3), it is possible that OXT anti-inflammatory and antioxidant effects stem from its influences on mitochondrial function (Boeck et al., 2018), and vice versa. Also considering that OXT regulates the stress response, there may be intricate interactions in which OXT indirectly affects mitochondria through its suppressive modulation of the HPA axis. Finally, OXT affects GABA signaling and the pattern of GABA_A_R subtype expression (Kaneko et al., 2016), which is related to its inhibitory effects on stress response (Levin and Ein-Dor, 2023). Thus, OXT may impact mitochondrial function through various interconnected mechanisms, which will be crucial to tease apart in the future.

Here we have outlined several key biological processes that take place during the peripartum period and are cited as potential etiological factors underlying peripartum disorders. We have described how mitochondria lie upstream of each of these physiological adaptations, highlighting the potential for mitochondrial origins in peripartum disorders. In situations where mitochondrial function is compromised, one or more of these processes may also be affected, leading to maladaptive physiology.

## 3 Evidence for mitochondrial links to maternal stress and mental health

### 3.1 Clinical studies in periphery

There is a dearth of studies investigating the role of mitochondria in the context of stress and maternal mental health. However, several groups have sought to determine mitochondrial links to maternal health and stress, which have contributed to the burgeoning understanding of the potential role of mitochondria in stress-related peripartum mood disorders such as PPD. The majority of these studies have been in the clinical setting and therefore report mitochondrial outputs mainly in blood (i.e., peripheral blood mononuclear cells – PBMCs, serum, and plasma) and placenta. Moreso, these studies focus primarily on stress history (for example, early life stress) and conclude more on offspring effects than the relationship of mitochondria to the mother’s mental health.

Recently, work from Kolassa’s group documented a relationship between maternal stress and mitochondria through multiple studies on a cohort of postpartum women with a history of childhood maltreatment. These studies overall demonstrated that women with a history of severe childhood maltreatment had higher mitochondrial respiration (correlating with severity of maltreatment) and higher mitochondrial density in PBMCs (immune cells) shortly after parturition (Gumpp et al., 2020, 2022), associated with higher inflammation and evidence of ROS. These effects were also detectable 3 months postpartum (Boeck et al., 2016, 2018). However, a later study demonstrated that group differences in mitochondrial measures were no longer apparent 1 year after parturition (Gumpp et al., 2022). Together, these findings suggest that women with a history of severe early life stress have a sensitized inflammatory response that may require a higher energy demand, evidenced by higher mitochondrial respiration and density (Gumpp et al., 2022). Interestingly, recent work by the Picard group revealed that chronic exposure to glucocorticoids similarly induced a hypermetabolic phenotype, with enhanced mitochondrial respiration in human fibroblasts that ultimately led to mtDNA instability, accelerated aging, and a reduced lifespan (Bobba-Alves et al., 2022). Thus, chronic stress exposure may exert lasting effects on metabolism that interact with parity to alter how mitochondria, at least within maternal peripheral cells, respond to an event as physiologically dramatic as birth.

Omics-based approaches (i.e., transcriptomics, proteomics, metabolomics) analyzing associations between maternal psychological symptoms and different biological pathways have provided complementary findings to the possibility of a mitochondrial role in maternal mental health. For example, Laketic and others recently sought to identify significant metabolic pathways associated with depression and anxiety symptoms in pregnant mothers at mid-gestation (28–32 weeks) (Laketic et al., 2023). Psychological symptoms were positively associated with different serum metabolites relevant to energy production, specifically alanine, leucine, phenylalanine, lactate, and glucose, which are involved in glycolysis and the TCA cycle (Laketic et al., 2023). The authors posit that such increases in these metabolites in depressed pregnant individuals may reflect an increase in energy demand (Laketic et al., 2023), which would align with the findings from Kolassa’s group as discussed above. Another group conducted transcriptomics (i.e. RNA-sequencing) on PBMCs of postpartum women, associating their gene profiles with symptoms of depression (Pan et al., 2018). PPD symptoms were positively correlated with multiple genes in energy metabolism, including OXPHOS, pyruvate metabolism, glycolysis/gluconeogenesis, ether lipid metabolism, and the TCA cycle (Pan et al., 2018). In a metabolomic assessment of serum samples in pregnant women who developed PPD, Papadopoulou et al. (2019) reported elevated glutathione-disulfide and ATP metabolites in PPD patients versus postpartum controls, which are associated with oxidative stress and energy production, respectively. In addition, this study reported an imbalance in the glutathione:glutathione-disulfide ratio, implying increased oxidative stress as glutathione-disulfide is the oxidized form of the antioxidant glutathione (Papadopoulou et al., 2019). This point is further supported by a 2017 study which reported an increase in indicators of oxidative and nitrosative stress and decrease in antioxidant markers in plasma of PPD patients (Roomruangwong et al., 2017a). Importantly, when imbalanced, such processes may damage mitochondria and ultimately affect function (Murphy, 2009). While the authors do not speculate on their finding of increased serum ATP (Papadopoulou et al., 2019), these omics-based findings altogether support the possibility that PPD is accompanied by an increased energy demand (Pan et al., 2018; Papadopoulou et al., 2019; Laketic et al., 2023). This alteration could result in mitochondrial dysfunction when mitochondrial allostatic load is reached and energy reserves are depleted (Morava and Kozicz, 2013; Picard and McEwen, 2018; Bobba-Alves et al., 2023).

Clinical studies are generally limited by tissue/sample type, though pregnancy comes with a unique temporary endocrine organ, the placenta, which has provided valuable information regarding the maternal-fetal environment. Like the brain, the placenta elicits high energy demands to function optimally (Armistead et al., 2020) and is affected by maternal stress. Lambertini et al. (2015) explored the relationship between maternal psychosocial stress during pregnancy and placental mitochondrial-encoded gene expression, showing that such genes are associated with maternal perceived stress scores. In particular, *MT-ND6*, a gene involved in encoding mitochondrial complex I, showed decreased expression with increasing stress scores. While respiratory function was not assessed, the authors speculate that deficiency in complex I gene expression is largely implicated in mitochondrial energy metabolism deficits since complex I is a major contributor to the proton flux which drives ATP synthesis (Wikström, 1984; Galkin et al., 2006; Kühn et al., 2015; Lambertini et al., 2015). Wright’s group has also focused on mitochondrial signatures within the placenta, specifically mtDNA copy number, which may be indicative of oxidative stress (Brunst et al., 2017). Though they assessed associations with both lifetime stress and prenatal stress, they found that higher lifetime stress events and psychological symptoms (i.e., depression or PTSD) were most predictive of reduced placental mtDNA copy number (Brunst et al., 2017). As physiological characteristics of the placenta widely affect maternal parameters (such as circulating hormone levels) (Hahn-Holbrook et al., 2013; Kikuchi et al., 2021), it is possible that such changes in placental mitochondrial-encoded gene expression and mtDNA copy number could have consequences on maternal mental health, and/or be reflective of mitochondrial dysfunction within mothers. Overall, these data indicate a likely role for mitochondrial involvement in responding to maternal psychosocial stress during pregnancy, though the question remains of how placental mitochondrial signatures may reflect maternal mitochondrial function, particularly in the brain.

### 3.2 Preclinical studies in rodent brain

While several groups have begun to elucidate mitochondrial links to maternal health and stress in periphery, studies in the brain have been largely neglected due to the difficulty of assessing brain mitochondrial function in clinical studies, though preclinical studies are also sparse. Recent work from our lab has been the first to demonstrate a direct link between gestational stress and brain mitochondrial function in rodents. We exposed pregnant and non-pregnant female rats to chronic mild unpredictable stress or handling and examined mitochondrial respiration, content, and complex protein levels in the mid-postpartum (Gorman-Sandler et al., 2023). Our stress exposure induced PPD-relevant behaviors in gestationally stressed female rats, including reduced maternal care and anhedonia. Importantly, we observed a significant reduction in mitochondrial respiration in the prefrontal cortex of gestationally stressed female rats compared to non-stressed postpartum counterparts (Gorman-Sandler et al., 2023). While there was no evidence of altered mitochondrial content, we did observe decreased complex I expression relative to non-stressed counterparts. These differences were accompanied by increased markers of neuroinflammation, but no alterations in ROS-induced lipid peroxidation products (Gorman-Sandler et al., 2023). This does not necessarily indicate *no* ROS production since protein oxidation has also been implicated in PPD (Roomruangwong et al., 2018). In the nucleus accumbens of the same animals, mitochondrial respiration and content were unaffected, demonstrating that mitochondrial respiratory function is not globally impacted by gestational stress. Previous work has implicated the prefrontal cortex in vulnerability to stress (Weger et al., 2020) and anxiety (Hollis et al., 2018) in male rodents, highlighting mitochondrial respiratory capacity in prefrontal cortex as an important factor in stress resilience.

In another recent study examining brain mitochondrial measures following maternal stress, Cruz et al. (2023) reported that offspring deprivation stress in postpartum mice, both alone and in combination with obesity (induced via high-fat diet), influenced TCA cycle and mitochondrial complexes I and II enzyme activity. Most susceptible to these treatments were the hippocampus and striatum, which had reduced activity of complexes I or II, respectively (Cruz et al., 2023). Interestingly, stressed female mice did not exhibit decreased complex I activity in the prefrontal cortex (Cruz et al., 2023), in contrast to our recent findings. However, it is important to note that the type and timing of stress exposures and species were significantly different (gestational chronic mild unpredictable stress in outbred rats vs. postpartum offspring deprivation in inbred mice). Such differing results highlight the importance of examining effects of stress on mitochondrial function across species, peripartum period, and stressor-type. Together, these studies suggest that both gestational and postpartum stressors can influence brain mitochondrial signatures and function, emphasizing the peripartum as an overall vulnerable period to stress (Cruz et al., 2023; Gorman-Sandler et al., 2023). However, whether certain windows of reproductive experience (i.e., early or late trimester, parturition, early or late postpartum, etc.) are more or less vulnerable to stress-induced mitochondrial alterations remains to be investigated. Clearly, much is to be understood regarding brain mitochondrial function in the context of peripartum stress and mood disorders.

The current evidence in the literature reliably shows a link between a history of perceived stress and mitochondrial features (summarized in Table 2). The findings from clinical studies suggest that a history of stress prior to gestation induces a hypermetabolic phenotype in the periphery associated with oxidative stress and inflammation. It should be noted that these studies are necessarily correlative in nature and consist of variable self-reported stress experiences rather than controlled manipulations. That mitochondrial signatures were detected in similar directions across studies despite this variability underscores the strength and size of this link with perceived stress. The preclinical findings in the brain further support a role for mitochondrial function in peripartum health, with stress decreasing mitochondrial complex I activity in specific brain regions (Cruz et al., 2023; Gorman-Sandler et al., 2023). It is interesting that peripheral mitochondrial measures point to a hypermetabolic state while neural measures suggest reduced capacity and further studies will be necessary to fully understand the relationship between peripheral and central mitochondrial function.

**Table 2:** Clinical and preclinical mitochondrial findings in peripartum stress literature.

**Study Subject**	**Sample(s) Measured**	**Mitochondrial Measure**	**Mitochondrial Outcome**	**References**
Clinical studies				
Postpartum women at delivery, evaluated for psychological stress during pregnancy	Placenta	Gene expression of 13 protein-coding genes encoded by the mitochondria	*MT-ND2* positively associated with indices of maternal psychosocial stress in pregnancy, while *MT-ND6* negatively associated	Lambertini et al. (2015)
Postpartum women with or without a history of childhood maltreatment, 3 months postpartum	Serum, PBMCs,	Metabolomics for markers of oxidative stress, respiration (Oxygraph) and density (citrate synthase activity)	↑ serum oxidative stress markers with higher maltreatment load, ↓ metabolites with antioxidative capacities, ↑ respiration with maltreatment load, no group differences in mitochondrial density, ↑ pro-inflammatory cytokines were positively associated with respiration and ROS production	Boeck et al. (2016)
Pregnant women evaluated for psychological stress and depression/PTSD symptoms	Placenta	mtDNA copy number (qRT-PCR)	Maternal psychosocial stress negatively associated with with placental mtDNAcn	Brunst et al. (2017)
Pregnant women at end of term, with or without prenatal depression	Plasma	Antioxidants and markers of oxidative stress, nitrosative stress	significant alterations of OS and NS with pregnancy, ↓ antioxidant defenses, ↑ OS and NS at the end of term is associated with perinatal depressive symptoms	Roomruangwong et al. (2017a,b)
Postpartum women with or without a history of childhood maltreatment, 3 months postpartum	PBMCs	Respiration (Oxygraph), density (citrate synthase activity)	Cortisol levels positively associated with mitochondrial respiration, but oxytocin levels negatively associated with respiration. These associations were only observed in women with high maltreatment load	Boeck et al. (2018)
Postpartum women following delivery, who did or did not develop PPD	PBMCs	Transcriptomics (RNA sequencing)	PPD symptoms positively associated with multiple genes involved in energy metabolism and immune response, serum concentrations of IL1β, CXCL2 and CXCL3 (cytokines and chemokines) ↑ in PPD groups	Pan et al. (2018)
Pregnant women at 14 weeks gestation, who did or did not develop PPD	Serum	Metabolomics (SRM-based mass spectometry)	↑ levels of glutathione-disulfide, adenylosuccinate, and ATP, which are related to oxidative stress, nucleotide biosynthesis and energy production pathways	Papadopoulou et al. (2019)
Postpartum women with or without a history of childhood maltreatment, within 1 week following parturition	PBMCs	Respiration (Oxygraph), density (citrate synthase activity)	↑ respiration and density in women with CM, CM load positively correlated with mitochondrial respiration	Gumpp et al. (2020)
Postpartum women with or without a history of childhood maltreatment, within 1 week following parturition and 1 year postpartum	PBMCs	Respiration (Oxygraph), density (citrate synthase activity)	↑ mitochondrial respiration in all women from week after parturition to 1 year postpartum, ↑ mitochondrial respiration and density in CM women at 1 week postpartum but no group differences 1 year postpartum	Gumpp et al. (2022)
Pregnant women between 28–32 weeks of gestation, evaluated for depression and anxiety symptoms	Serum	Metabolites (H-NMR) and micronutrients (ICP-MS)	Depression scores had positive associations with alanine, glucose, lactate, leucine, methionine, phenylalanine, pyruvate, valine, 3-hydroxybutyrate, and antimony	Laketic et al. (2023)
Preclinical studies				
Female adult Swiss mice, fed a high fat diet for 8 weeks pre-pregnancy which continued until 21 days postpartum, and/or offspring deprived for 10 days postpartum	Brain Tissue (PFC, HTL, Striatum, and Hipp)	Krebs cycle enzyme activity, mitochondrial OXPHOS complexes activity, creatine kinase activity	Brain regions where mitochondrial enzyme activities were most affected by obesity and offspring deprivation were the HTL and Hipp	Cruz et al. (2023)
Female adult Wistar rats, stressed during gestation and euthanized at 11-12 days postpartum	Brain tissue (PFC and NAc), plasma	Respiration (Oxygraph), protein expression of OXPHOS complexes and 4-HNE (marker of oxidative stress)	↓ mitochondrial respiration in PFC, ↓ complex I protein expression in PFC, no group differences in 4-HNE protein expression in PFC, ↑ pro-inflammatory cytokines in plasma and PFC, negative correlation between TNF-α and resipration in PFC	Gorman-Sandler et al. (2023)

Abbreviations: PBMCs, peripheral blood mononuclear cells; PFC, prefrontal cortex; HTL, hypothalamus; Hipp, hippocampus; NAc, nucleus accumbens, SRM, selected reaction monitoring; H-NMR, proton nuclear magnetic resonance; ICP-MS, inductively coupled plasma-mass spectrometry.

## 4 Potential role for mitochondria in stress resilience to PPD pathology

**Figure 2:**
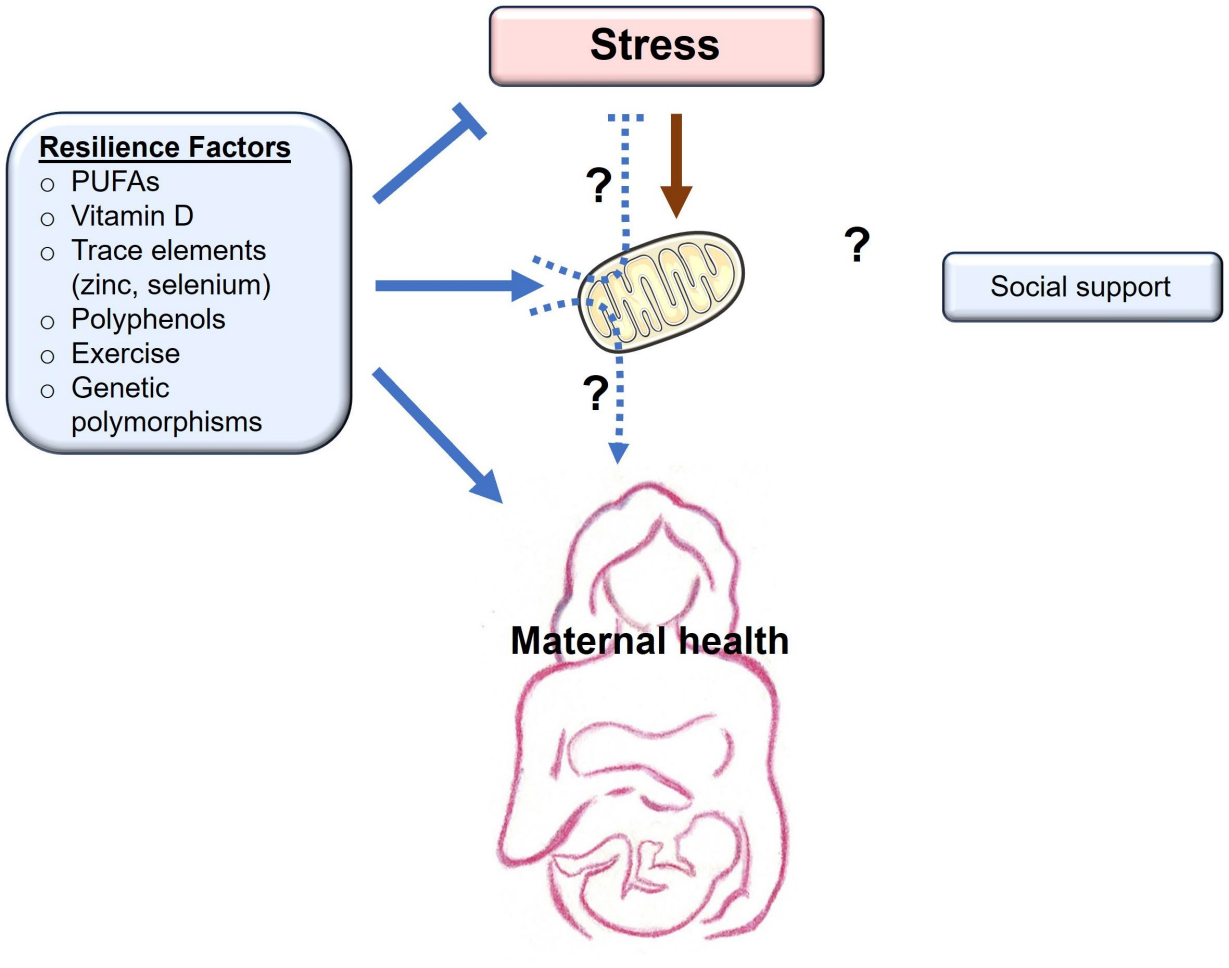
Resilience factors buffer the effects of stress on maternal health. Chronic stress exerts negative (orange arrows) effects on mitochondrial function, with detrimental consequences to maternal health. Resilience factors, including PUFAs, vitamin D, trace elements, polyphenols, exercise, and genetic polymorphisms, all buffer stress, promote (blue arrows) mitochondrial health, and are associated with improved maternal health. Whether the positive effects of resilience factors occur as a result of their beneficial effects on mitochondrial function remains to be determined (dashed arrows). Social support is another resilience factor that buffers the effects of stress and promotes maternal health. Whether social support has beneficial effects on mitochondrial function remains to be determined (dashed arrows).

While previous studies have established a link between gestational stress and the onset of PPD, not everyone who experiences stress in the peripartum will develop PPD. This implies that there are underlying resilience factors that influence the development of the condition. While many studies focus on the role psychosocial and cultural factors play in the prevention of PPD, other studies in both clinical and preclinical settings have assessed varying environmental resilience factors such as diet, nutrition, and exercise. The biological mechanisms behind such resilience factors have not been explored in depth, and mitochondria may be potential mediators underlying these resilience-boosting effects (Figure 2).

### 4.1 Nutritional status

Pregnant and lactating women are at a higher risk for suboptimal nutritional status as the demands required for these events are higher than when not pregnant or lactating (Bodnar and Wisner, 2005). Diet and nutrition are modifiable factors that may incur resilience against developing PPD, while poor nutrition may increase risk for pregnancy complications or pregnancy-related disorders (Bodnar and Wisner, 2005; Leung and Kaplan, 2009). Mitochondria are key components of metabolism and are closely linked to nutrition, as many nutrients and trace elements act as cofactors or antioxidants to ultimately promote mitochondrial function (Wesselink et al., 2019; Rodríguez-Cano et al., 2020). Thus, a healthy diet with proper nutrition and intake of prenatal/postnatal vitamins may contribute to resilience in the face of stressors by manipulating mitochondrial function.

#### 4.1.1 Polyunsaturated fatty acids

Polyunsaturated fatty acids (PUFAs) are critical for growth and maturation of fetal organs, with the omega-3 PUFAs, such as docosahexaenoic acid (DHA), being especially important for fetal brain development during late pregnancy and early infancy (Al et al., 2000) and for maternal brain health (Dighriri et al., 2022). Several studies have implicated low peripheral omega-3 PUFAs in risk for prenatal and postpartum depression (Levant, 2011; Lin et al., 2017), and diets rich in DHA (ex. seafood) are associated with resilience against development of PPD (Hibbeln, 2002). Fish oils rich in DHA and other PUFAs are shown to improve mitochondrial function, affecting ROS production, bioenergetics, membrane potential, dynamics, and biogenesis (Afshordel et al., 2015; Putti et al., 2015; de Oliveira et al., 2017). Similar to these studies, adherence to the Mediterranean diet, which naturally involves higher consumption of omega-3 PUFAS, increased resilience against PPD (Flor-Alemany et al., 2022). Like omega-3 s, the Mediterranean diet can also reduce mitochondrial ROS, protecting against oxidative stress and apoptosis (Pollicino et al., 2023). Meanwhile, rodent models of obesity such as a high-fat diet have been linked to poor maternal behavior (Bellisario et al., 2015; Bolton et al., 2017), negative impacts on brain and spinal cord mitochondrial function, and other parameters that affect mitochondria such as ROS production and fission (Langley et al., 2020; Cruz et al., 2023). In humans, a higher ratio of n-6 to omega-3 PUFA consumption has been associated with depression and PPD (Bodnar and Wisner, 2005). This is an interesting concept, as the Mediterranean diet is characterized by a good balance of n-6 to omega-3 PUFA intake, while more Westernized and high-fat diets involve much higher intake of n-6 PUFAs (Mariamenatu and Abdu, 2021). PUFAs also have additional immunomodulatory and neuroplastic properties, which may be related to their effects on mitochondrial function as discussed in previous sections (Su et al., 2015; Pérez et al., 2018; Abdul Aziz et al., 2021).

#### 4.1.2 Vitamin D

While the consensus is somewhat mixed, meta-analyses and reviews overall suggest a relationship between low vitamin D levels and higher depressive symptoms, and that vitamin D supplementation may reduce risk for prenatal and postnatal depression (Trujillo et al., 2018; Dowlati and Meyer, 2021; Rupanagunta et al., 2023). Vitamin D is a point of interest due to its potential role in mitochondrial function, where vitamin D and its receptor modulate mitochondrial respiratory chain activity. For example, Ricca et al. (2018) demonstrated that silencing of the vitamin D receptor gene in different cell types led to increased mitochondrial respiration with corresponding excessive production of intracellular ROS, resulting in oxidative damage and eventual apoptosis. Thus, vitamin D influences mitochondrial activity and protects cells from oxidative stress and cytotoxicity (Consiglio et al., 2015; Ricca et al., 2018). Vitamin D may also rescue mitochondrial respiration in cases where it is decreased, such as in trophoblasts from placental tissue of obese mothers (Phillips et al., 2022).

#### 4.1.3 Minerals

Trace elements such as zinc and selenium have also been implicated in PPD, where zinc supplementation has shown to have buffering effects against gestational stress (Roy et al., 2010) and protective effects against development of PPD (Aoki et al., 2022), while low zinc levels are predictive of depressive symptoms in the postpartum (Roomruangwong et al., 2017b). Dietary zinc can prevent ROS accumulation and decreases in antioxidant activity (Zhou et al., 2005), and increase mitochondrial electron transport system enzyme activity (Corona et al., 2010). Although a proper balance seems important as excess zinc can disrupt mitochondrial membrane potential, increase ROS, and inhibit mitochondrial respiration (Dineley et al., 2005; Liu et al., 2021). Similarly, selenium supplementation during pregnancy reduced the risk for onset of PPD (Mokhber et al., 2011), an effect which could be related to mitochondrial influences as selenium can promote mitochondrial dynamics and respiratory capacity, restore membrane potential, and decrease ROS production (Chen et al., 2021; Wesolowski et al., 2022).

#### 4.1.4 Polyphenols

Polyphenols are compounds largely found in fruits and vegetables that have antioxidant, anti-inflammatory (Nacka-Aleksić et al., 2022), and mood-regulatory properties. For example, two-week consumption of a high-flavonoid diet during the postnatal period reduced anxiety and increased perceived quality of life in mothers (Barfoot et al., 2021). Flavonoids protect cells against insult through effects on mitochondrial dynamics, biogenesis, and mitophagy (Kicinska and Jarmuszkiewicz, 2020). Resveratrol, a non-flavonoid polyphenol, has been used as a pharmacological agent in rodent models of PPD (Ye et al., 2023), and could promote resilience through its indirect activation of sirtuin 1 (Beher et al., 2009) and downstream effects on transcriptional coactivators that stimulate mitochondrial biogenesis (Lehman et al., 2000; Higashida et al., 2013; Zhou et al., 2018; Majeed et al., 2021), though currently no clinical studies exist investigating the therapeutic potential of resveratrol for peripartum mental health. The influence of resveratrol on mitochondrial biogenesis (Lagouge et al., 2006), in addition to having anti-inflammatory and antioxidant effects (Chen et al., 2017; Finnell et al., 2017), could affect mitochondrial function through multiple mechanisms. Thus, while polyphenols have not been explored in depth in the context of peripartum mood disorders, their potential to confer resilience seems promising as suggested by studies in clinical instances and preclinical models of major depression (Lin et al., 2021; Gamage et al., 2023).

### 4.2 Exercise

Exercise and physical activity have long been studied in the context of stress-related psychiatric disorders (Ströhle, 2009), and have largely shown promise as a relatively safe and cost-effective non-pharmacologic intervention for PPD (Kołomańska-Bogucka and Mazur-Bialy, 2019; Marconcin et al., 2021), though effectiveness may depend on length of physical activity and regimen (Yuan et al., 2022). For example, yoga is widely regarded as an effective adjunctive treatment for chronic disease because of its extensively researched benefits. A study conducted by Buttner et al. (2015) affirmed yoga’s potential to alleviate symptoms associated with PPD, as women who underwent yoga therapy reported significantly lower levels of depression and anxiety than control groups. While yoga has various established physiological effects, it also has lesser-known effects on mitochondrial parameters (Gautam et al., 2021b). One study showed that 8 weeks of yoga intervention (which included breathing techniques, physical postures, and meditation) in rheumatoid arthritis patients reduced peripheral ROS while bolstering peripheral markers of mitochondrial integrity including sirtuin 1 mRNA expression, total antioxidant capacity, membrane potential, mtDNA copy number, and activity of nicotinamide adenine dinucleotide (NAD+) and cytochrome c oxidase-II (Gautam et al., 2021a). Though mitochondrial respiration was not assessed, these parameters all suggest that yoga has the potential to improve mitochondrial function (Gautam et al., 2021a).

Rodent models have offered further insight into the effects exercise may have on brain mitochondria in the wake of chronic stress, as rodent offspring exposed to prenatal or early life stress demonstrate alterations in brain mitochondrial morphology, function, mRNA and protein expression, and oxidative stress that are reversed by treadmill exercise (Park et al., 2019; Wu et al., 2019). Thus, despite the additional influences exercise and physical activity have on inflammation (Liu et al., 2013), HPA axis activity (Wu et al., 2019), and neuroplasticity (Aguiar et al., 2014), it is possible that their mitochondrial-enhancing qualities ultimately drive the protective effects on stress-related behaviors and PPD.

### 4.3 Genetics

So far, we have considered environmental influences on resilience, however, the importance of genetic factors cannot be overstated. Indeed, the mitochondrial genome has a high rate of mutagenesis due to its exposure to external and internal noxious stimuli (Alexeyev et al., 2013). This leads to frequent mutations that may affect mtDNA stability and confer susceptibility to further challenges such as chronic stress (Bobba-Alves et al., 2023). Additionally, while genetic association studies in PPD are scarce, a few notable associations have been identified with mitochondrial interactors. Genetic polymorphisms for estrogen signaling, monoamine-related, glucocorticoid and corticotropin releasing hormone genes have all been associated with increased development of PPD (Costas et al., 2010; Engineer et al., 2013; Rupanagunta et al., 2023). Similarly, there are few twin studies examining the heritability of PPD. Current studies report a heritable component, but suffer from limitations due to retrospective reviews, failure to control for comorbid diseases, and failure to account for shared environmental influences (Corwin et al., 2010). Interestingly, patients with mitochondrial disease do not necessarily exhibit higher rates of depression and anxiety compared to general population, though symptoms may intensify during pregnancy (Karaa et al., 2019). Future studies examining specific mitochondrial polymorphisms and/or genetic instability may shed further light on potential mitochondrial genetic origins of PPD.

### 4.4 Outstanding questions regarding mitochondria in resilience

Overall, we propose that the environmental and genetic factors known to promote resilience against PPD and PPA could do so through enhancing mitochondrial function. This may be accomplished either directly through actions on mitochondrial membrane potential, dynamics, bioenergetics, and biogenesis, or less directly through immunomodulatory and antioxidant effects. Indeed, the effects of diet, nutrition, exercise, and genetics on resilience to perinatal mental health should be further investigated with emphasis on whether the mechanisms are solely mitochondrial-dependent, or rather multifaceted. Moreso, combination therapies should be explored (Dowlati and Meyer, 2021), as monotherapies are less representative of the multi-nutritional profile of everyday diets and may be less effective. For example, response to antidepressants can depend on factors such as nutritional status, supporting the notion that adjunct supplementation may be more effective than either treatment alone (Bodnar and Wisner, 2005). Additionally, as it is difficult to tease apart pre-disposing resilience factors from passive resilience (i.e., where “resilience” is due to a lack of a marker in susceptible subjects, such as nutrient deficiency) (Pfau and Russo, 2015), the field should shift focus to understanding whether and how pre-existing individual differences play a role in resilience to PPD. Finally, as psychological and socioeconomic resilience factors are some of the most studied in the clinical setting (Julian et al., 2021; Alves et al., 2023), we must aim to better understand how these factors are related to biology, and specifically how they are related to mitochondrial function. After all, preclinical work demonstrates that mitochondria are involved in social behaviors and may be affected by social stressors (Hollis et al., 2015, 2022; Ülgen et al., 2023), suggesting that these factors are intertwined with biology. Thus, it is possible that such psychological, socioeconomic, sociocultural, and other social factors interact with stress resilience or vulnerability due to effects on mitochondria (Picard and McEwen, 2018). This field is largely unexplored apart from recent work by Wright’s group reporting differences in mtDNA mutations between mothers of different ethnic backgrounds, where women of black and African ancestry may be more vulnerable to mtDNA placental mutations (Brunst et al., 2017, 2021, 2022; Cowell et al., 2021).

## 5 Conclusion and future directions

**Figure 3:**
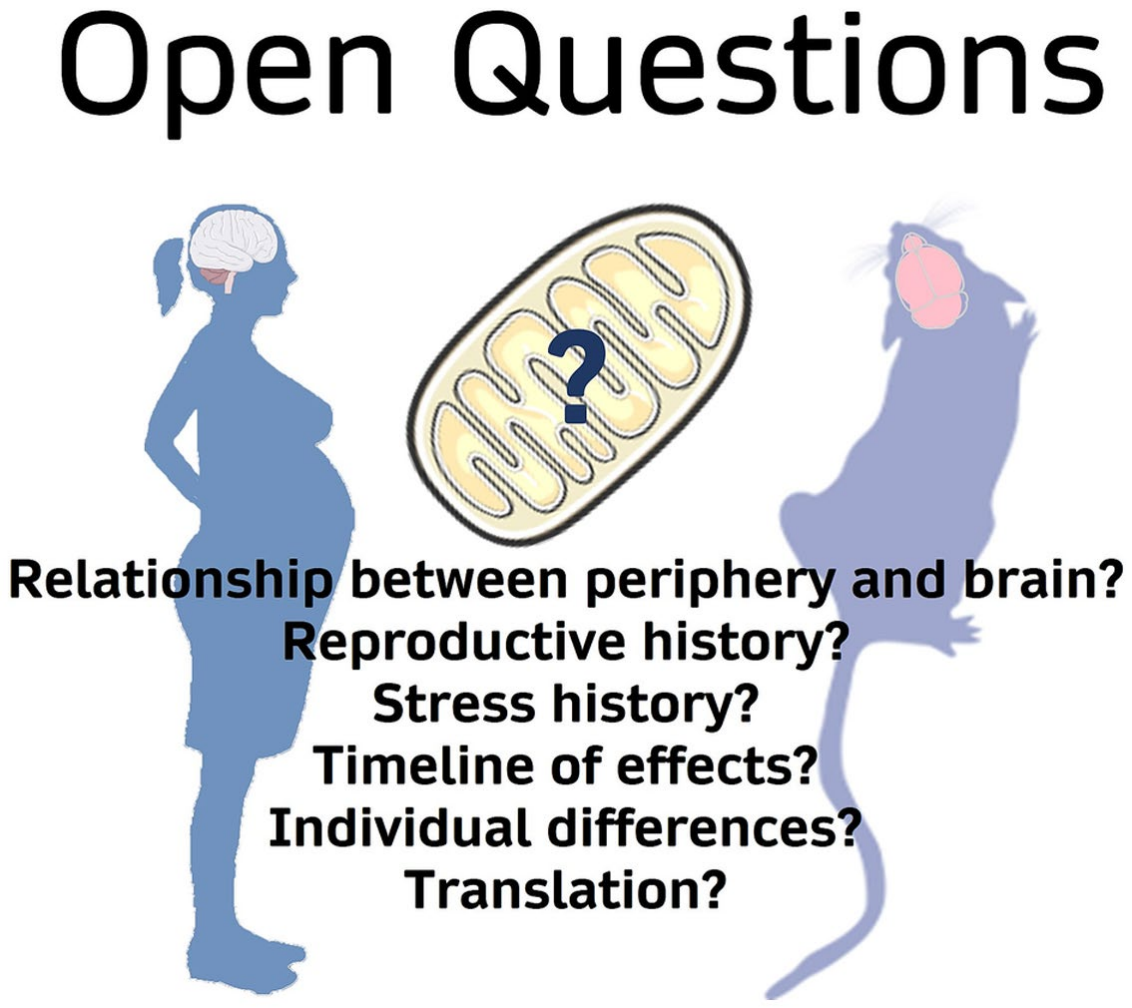
Open questions surrounding mitochondrial function in peripartum stress research.

Female health has long been underprioritized and therefore understudied, though a recent push for understanding sex as a biological variable has begun to shift the focus of psychiatric research (Galea and Parekh, 2023). While this shift is beginning to reveal the importance of sex differences in biology and behavior, reproductive experience remains a largely neglected area, despite most women undergoing pregnancy and parturition at least once in their lives (Martinez et al., 2018). Here we have presented the intimate links that mitochondrial function has with several of the key physiological adaptations occurring in the peripartum. Our understanding of mitochondrial function across the peripartum period, however, remains incomplete, especially in the brain – an organ exquisitely sensitive to metabolic fluctuations (Figure 3).

Future research should consider interactions between mitochondrial function and reproductive history. Whether parity exerts a lasting effect on mitochondrial function – and whether this signature is similar across the brain and periphery is unknown. A number of important works have described parity-induced alterations in the brain lasting beyond delivery and even weaning (reviewed in Puri et al., 2023). Indeed, reproductive events have been found to alter mitochondrial capacity in the liver as well as organ-specific responses to oxidative stress (Wood et al., 2019; Park et al., 2020). It is therefore tempting to speculate that altered brain mitochondrial function might underlie or associate with these adaptations. Furthermore, whether peripheral or placental mitochondrial signatures are reflective of maternal brain mitochondrial function and mental health is a crucial area of research as this could generate biomarkers for postpartum health. Recent work uncovered an association between aberrant circulating DNA, including *cf*-mtDNA, and a clinical diagnosis of pre-eclampsia (Cushen et al., 2022). Additional studies that examine mitochondrial dynamics during pregnancy may identify further associations.

Additional research is needed at the intersection between stress and parity. Our work shows that gestational stress can induce brain region-specific alterations in mitochondrial respiration and protein expression in the mid-postpartum period. However, the duration of these alterations and whether they confer susceptibility to later-life pathologies are open questions. The timing and intensity of stressors are additional areas of interest. We examined the effects of a 10-day chronic mild unpredictable stress exposure, though shorter or longer exposures of varying intensities may incur lesser or greater energetic consequences. The period of exposure may play a role in their effects, as there are several rodent protocols of gestational stress that are presented during differing gestational windows (Table 1). Additionally, there may be distinct contributions of prenatal stress, early life stress, or overall “lifetime stress” from pregnancy and postpartum stress (Brummelte and Galea, 2016). Given the number of physiological adaptations occurring across the gestational period, it is unsurprising that stress exposures during particularly energetically costly periods could have more significant consequences.

Finally, individual differences in response to stress are especially important to consider for identifying future therapeutic targets. We’ve previously shown significant natural variation in brain mitochondrial function linked to behavior in male rodents (Hollis et al., 2015, 2018; van der Kooij et al., 2018). Moreover, recently published data show variations in mitochondrial responses to chronic stress in male mice (Rosenberg et al., 2023). Thus, individual differences in mitochondrial responses to stress in females are likely and may interact with gestational physiological adaptations to generate unique patterns of resiliency and/or susceptibility. Indeed, recent reports indicate that parity alone induces individual differences in measures of anhedonia such as sucrose preference (Gifford et al., 2023). Variations in mitochondrial responses to stress could contribute to divergent treatment efficacy. Thus, studies should make sure to power for the analysis of individual differences. Mitochondria are emerging as critical mediators of health and disease. Here we have shown links between mitochondrial function and the physiological adaptations occurring across the peripartum period that may confer susceptibility to stress. Studies focused on delineating the role of mitochondrial function in the individual response to stress during this developmental period will provide valuable mechanistic insights and potentially new therapeutic targets for peripartum-associated neuropsychiatric diseases.

## Author contributions

EG-S: Conceptualization, Writing - original draft, Writing - review & editing. GW: Conceptualization, Writing - original draft, Writing - review & editing. NC: Visualization, Writing - review & editing. NF: Visualization, Writing - review & editing. HB: Visualization, Writing - review & editing. BR: Writing - review & editing. FH: Conceptualization, Funding acquisition, Supervision, Writing - original draft, Writing - review & editing.

## Figures and Tables

**Table:** 

ATP	adenosine triphosphate
*cf*-mtDNA	cell free mitochondrial DNA
DHA	docosahexaenoic acid
E2	estradiol
E3	estriol
GABA	gamma-aminobutyric acid
GABA_A_R	GABA A-type receptors
HPA	hypothalamic–pituitary–adrenal
mtDNA	mitochondrial DNA
n-6	class of polyunsaturated fatty acids
OXPHOS	oxidative phosphorylation
OXT	oxytocin
PBMCs	peripheral blood mononuclear cells
P4	progesterone
PPA	postpartum anxiety
PPD	postpartum depression
PUFAs	polyunsaturated fatty acids
ROS	reactive oxygen species
TCA	tricarboxylic acid (TCA)
TSPO	translocator protein (18 kDa)
